# A Novel Two-Step Method for Screening Shade Tolerant Mutant Plants via Dwarfism

**DOI:** 10.3389/fpls.2016.01495

**Published:** 2016-10-03

**Authors:** Wei Li, Lorenzo Katin-Grazzini, Sanalkumar Krishnan, Chandra Thammina, Rania El-Tanbouly, Huseyin Yer, Emily Merewitz, Karl Guillard, John Inguagiato, Richard J. McAvoy, Zongrang Liu, Yi Li

**Affiliations:** ^1^Department of Plant Science and Landscape Architecture, University of Connecticut, Storrs, CTUSA; ^2^Department of Crop Science, Michigan State University, East Lansing, MIUSA; ^3^Department of Floriculture, Ornamental Horticulture and Landscape Gardening, Faculty of Agriculture, Alexandria University, AlexandriaEgypt; ^4^Appalachian Fruit Research Station, United States Department of Agriculture, Agricultural Research Service, Kearneysville, WVUSA

**Keywords:** mutation breeding, screening method, dwarfism, gibberellins, shade tolerance

## Abstract

When subjected to shade, plants undergo rapid shoot elongation, which often makes them more prone to disease and mechanical damage. Shade-tolerant plants can be difficult to breed; however, they offer a substantial benefit over other varieties in low-light areas. Although perennial ryegrass (*Lolium perenne* L.) is a popular species of turf grasses because of their good appearance and fast establishment, the plant normally does not perform well under shade conditions. It has been reported that, in turfgrass, induced dwarfism can enhance shade tolerance. Here we describe a two-step procedure for isolating shade tolerant mutants of perennial ryegrass by first screening for dominant dwarf mutants, and then screening dwarf plants for shade tolerance. The two-step screening process to isolate shade tolerant mutants can be done efficiently with limited space at early seedling stages, which enables quick and efficient isolation of shade tolerant mutants, and thus facilitates development of shade tolerant new cultivars of turfgrasses. Using the method, we isolated 136 dwarf mutants from 300,000 mutagenized seeds, with 65 being shade tolerant (0.022%). When screened directly for shade tolerance, we recovered only four mutants from a population of 150,000 (0.003%) mutagenized seeds. One shade tolerant mutant, *shadow-1*, was characterized in detail. In addition to dwarfism, *shadow-1* and its sexual progeny displayed high degrees of tolerance to both natural and artificial shade. We showed that endogenous gibberellin (GA) content in *shadow-1* was higher than wild-type controls, and *shadow-1* was also partially GA insensitive. Our novel, simple and effective two-step screening method should be applicable to breeding shade tolerant cultivars of turfgrasses, ground covers, and other economically important crop plants that can be used under canopies of existing vegetation to increase productivity per unit area of land.

## Introduction

Perennial ryegrass (*Lolium perenne* L.) is a cool-season turfgrass that is widely used across the world (Jiang and Huang, 2001; Grinberg et al., 2016). It can be found in all manner of ornamental contexts, from small domestic lawns to sprawling golf courses. Perennial ryegrass is valued for its adaptability to a wide range of soil types, dark green color, and fast establishment (Hannaway et al., 1999; Cen et al., 2016). It is also popular as a forage crop, as it can easily tolerate repeated defoliation from grazing animals (Wilkins, 1991; Pearson et al., 2011). Perennial ryegrass germinates quickly under shade but plant health subsequently deteriorates. Under shade, other turfgrass species are recommended over perennial ryegrass, such as hard fescue (*Festuca brevipila*) and Supina bluegrass (*Poa supina*), due to their improved shade tolerance (Stier, 1999).

There are a number of techniques for creating new plant traits, ranging from traditional breeding to genetic engineering. Genetic engineering is the most powerful and effective way of introducing new traits to plants but there are concerns regarding the undesirable spread of transgenes through pollen and seeds (Hu et al., 2006; Kausch et al., 2010; Khan et al., 2016). Mutation breeding, one form of traditional breeding, can be effective in creating new plant traits without gene flow concerns, and may be useful for developing shade tolerance (Ahloowalia and Maluszynski, 2001; Shu et al., 2012). In the turf industry, shade tolerance is most relevant at mature stages, it is therefore preferable to screen mature, mutagenized plant populations for shade tolerance. However, direct screening for shade tolerance in mature turf can be cumbersome because of the time and space required.

In response to shade stress, perennial ryegrass becomes etiolated; possessing chlorotic, elongated leaf and shoot tissues, as a mechanism for seeking sunlight (Jiang et al., 2004; Faigón-Soverna et al., 2006). This condition is particularly detrimental in mowed turfgrass swards, where etiolation results in significant removal of leaf and stem tissue, depleting available carbohydrate reserves, and contributing to the decline of turfgrass sward health and appearance. Moreover, etiolation is associated with increased vulnerability to disease (Turgeon, 1991). As such, shade tolerant plants may be identified by a de-etiolated phenotype under low-light conditions.

Increased cell elongation is a key symptom of shade stress, and is especially detrimental under mowed conditions where etiolation results in excessive tissue removal (Casal, 2012). It is possible that inhibition of cell elongation in stems and leaves could conserve carbohydrate and chlorophyll reserves in these tissues, resulting in tolerance to low-light conditions. The reduced growth exhibited by dwarf perennial ryegrass mutants could make these plants shade tolerant, however, there is a lack of research exploring the influence of dwarfism-associated reductions in cell elongation on shade tolerance. It has been reported that chemically induced dwarfism improves the performance of turfgrass in the shade (Ervin et al., 2002; Goss et al., 2002; Studzinska et al., 2012), providing circumstantial evidence that some dwarf mutants could be shade tolerant. Identifying dwarfism is simple at the early stages of seedling development, making it possible to be done in a relatively small space over a short time frame. It has been well documented that many dwarf mutants are dominant or semi-dominant (Busov et al., 2008). Dwarfism itself is an important trait for perennial ryegrass, as it can reduce costs associated with mowing, irrigation, and fertilization (Hanna and Elsner, 1999; Lu et al., 2008, 2009). Other beneficial traits, such as prostrate growth, are also associated with dwarfism in perennial ryegrass (Chen et al., 2016).

In this manuscript, we describe the successful application of a two-step screening method, first for dwarfism and then for shade tolerance, to identify shade tolerant mutants of perennial ryegrass. Shade tolerant crop plants are highly valuable for enhancing agricultural productivity because they can be planted underneath shade intolerant crops for enhancing agricultural productivity on per unit area of land. We further show that one of the dwarf mutants that we isolated, *shadow-1*, is partially GA insensitive and is significantly more tolerant to high-intensity, long-term shade compared to its wild-type counterpart.

## Materials and Methods

### Two-Step Screening Procedure

**FIGURE 1:**
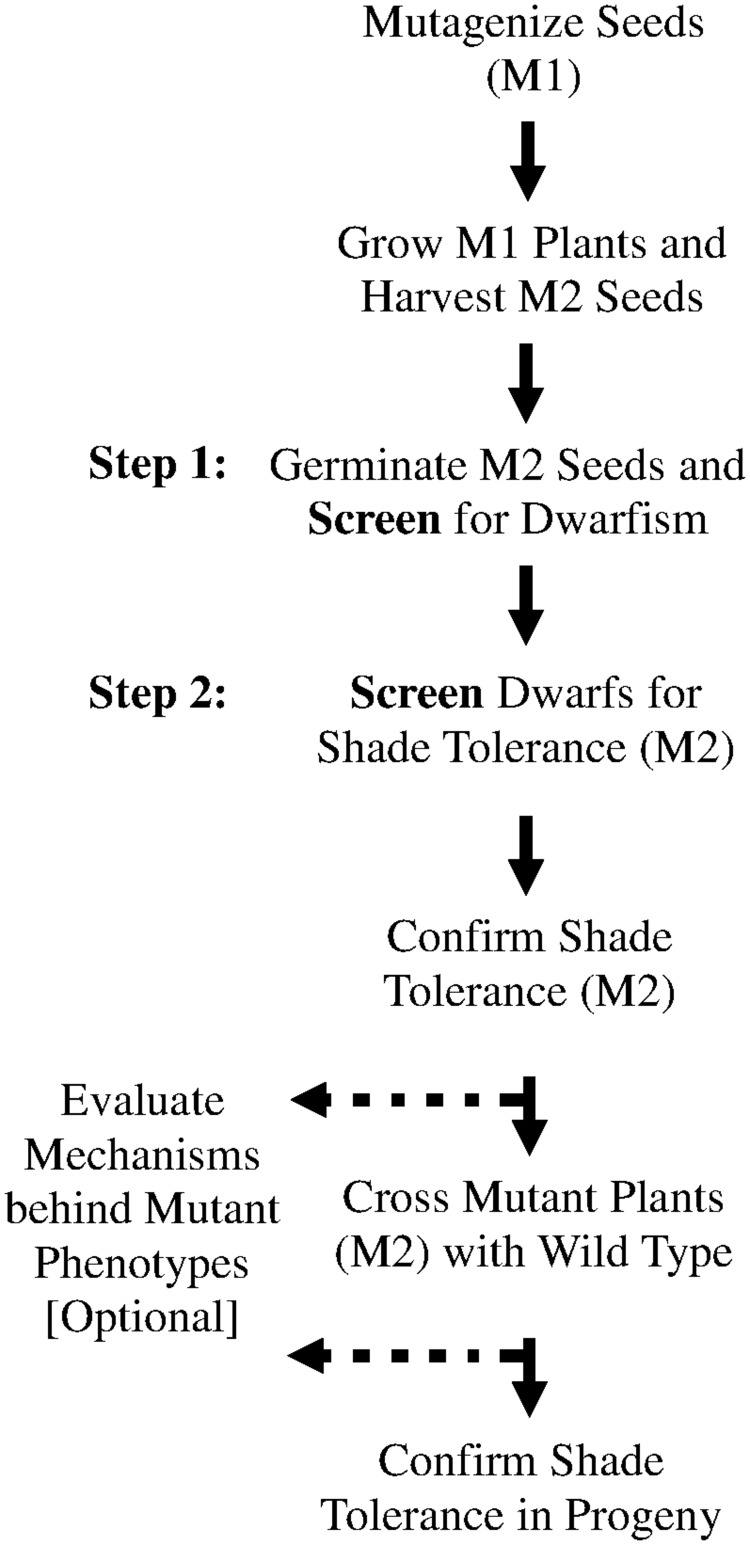
**Two-step screening can identify dominant shade tolerant mutants of perennial ryegrass.** Optional steps (dashed lines) consist of the elucidation of possible mechanisms behind the phenotypes observed in M2 plants.

**Figure 1** outlines the overall procedure of a two-step screening method. The two-step screening method first screens for dominant dwarf mutants at the three-leaf stage (Step 1) and then screens mature dwarf plants for shade tolerance (Step 2).

### Gamma-ray Irradiation and Producing M2 Seeds for Screening

Ten kilograms of ‘Fiesta 4’ perennial ryegrass seeds (DLF Pickseed USA, Tangent, OR, USA) were submerged in water for 24 h and then subjected to gamma-ray irradiation (9.0 kr) from a Cobalt-60 source in the Radiation Laboratory at the University of Massachusetts, Lowell, MA, USA. The irradiated seeds made up the M1 population and were air-dried at room temperature for 12 h and then stored at 4°C. M1 seeds were hand-broadcasted at a density of 1.5 kg per 100 m^2^ and plants were allowed to cross-pollinate randomly. M2 seeds were harvested, left to air-dry at room temperature for 12 h, and finally storedat 4°C.

### Direct Screening for Shade Tolerance

One hundred and fifty thousand M2 seeds were treated with 289 μM gibberellic acid for 10 h in order to encourage uniform germination. The seeds were rinsed and kept at 4°C for 2 weeks. Seeds were germinated in Pro-Mix potting soil (Premier Horticulture Inc., Quakertown, PA, USA) on 0.162 m^2^ trays under a frame covered in black polyfiber cloth which blocked 95% of sunlight (~600 lux on a sunny day verified with a UEi DLM2 Light Meter) in the greenhouse. After 2 weeks, seedlings were screened for shade tolerance. Shade tolerance was determined by a lack of etiolation, which was defined as short coleoptiles, emergent true leaves, and an overall reduction in seedling height. Putative shade-tolerant seedlings were then transferred to 50-plug trays (28 cm × 56 cm) and allowed to grow under full light in the greenhouse for 6 weeks. To confirm shade tolerance, putatively shade-tolerant plants were placed back under 95% shade for 6 weeks, alongside wild-type plants. Over this period wild-type plants were severely damaged and shade tolerance was confirmed by the healthy growth of mutant plants.

### Two-Step Screening for Dwarfism and Shade Tolerance

One hundred and fifty thousand M2 seeds were germinated as described above. Seedlings were planted in Pro-Mix soil and were kept under full light in the greenhouse. At the three-leaf stage, seedlings were screened for dwarfism, defined by a ≥30% reduction in leaf height, and dwarf seedlings were transferred to 50-plug trays. Seedlings were allowed to grow in full light for 6 weeks after which dwarf mutants were placed under 95% shade alongside wild-type plants for 6 weeks. Over this period wild-type plants were severely damaged and shade tolerance was confirmed by the healthy growth of mutant plants. One plant was selected for further evaluation and was given the name *shadow-1*.

### Greenhouse and Field Evaluation of *shadow-1* M2 Plants under Full Light

*shadow-1* and wild-type plants were vegetatively propagated by cutting the roots and shoots to a 2.5 cm length to insure uniformity, after which two tillers were placed in each plug of a 50-plug tray containing Pro-Mix soil. After growing for 2 months, plants were photographed and height data were taken. Six plugs of 2-month-old *shadow-1* and wild type were transferred to the field in September of 2012 after which they were fertilized and irrigated as needed. Canopy height was measured and photographs were taken on May 13, 2013, after which they were removed from the field. The plants were then dried at 70°C for 10 days. Dry plants were cut below the crown so that the roots and shoots of each plant could be weighed separately. Data were reported as the mean of the six replicates. Comparisons of means between *shadow-1* and wild-type data collected from greenhouse- and field-grown plants were conducted using two-tailed Student’s *t*-test with the pooled variance (Steel et al., 1997).

### Evaluation of *shadow-1* M2 Plants under 95% Shade in Greenhouse

*shadow-1* and wild-type plants were vegetatively propagated in rectangular pots (15 cm × 11 cm × 5 cm). Plants were first cut to a 2.5 cm root and shoot length, and six groups (two tillers each) were evenly spread within each of six pots for both *shadow-1* and wild type. Plants were maintained at a 5 cm height in full light for 6 weeks and then placed in a 95% shade environment (~600 lux on a sunny day verified with a UEi DLM2 Light Meter) in the greenhouse which was created by the use of black polyfiber cloth. Leaf lengths were recorded after 3 weeks of shade treatment as the average within each pot. These lengths were then combined and averaged between the six replicates.

### Evaluation of *shadow-1* M2 Plants under 95% Shade and 85% Shade in Field

*shadow-1* and wild-type plants were propagated in 50-plug trays as described above and were subsequently allowed to grow in full light for 6 weeks. Six plugs from both *shadow-1* and wild type were planted in the field at the beginning of September in 2012 and 2013, in a wooded area where shade was measured to be, on average, a 95% reduction in full light. In late May, 2013 and 2014, plants were cut to 5 cm and maintained at that height for the next 7 weeks (plants were mowed to 5 cm whenever they reached a height of 7.5 cm). At the beginning of July in each year, plants were dug up from the field and their tiller numbers were counted. Plants were then left to dry at 70°C for 10 days after which plants were cut below the crown and the dry root mass was weighed. Data were reported as a mean of the six replicates. Analysis of variance was performed on data collected 2013 and 2014 using IBM SPSS 19.0 (IBM Corp., Somers, NY, USA). When sufficient differences (*P* < 0.05) were observed, Fisher’s protected least significant difference test (*P* = 0.05) was performed to calculate differences between treatments.

Six *shadow-1* and wild type plugs were planted in a wooded area on May 10, 2013, where shade was measured to be a ~85% reduction in full light. The plants were left in that area and a representative replicate was photographed on June 11, 2013 and again on October 24, 2015.

### Evaluation of *shadow-1* Progeny Plants for Dwarfism and Shade Tolerance

*shadow-1* and wild-type plants were vegetatively propagated in 50-plug trays as described above. On September 25, 2012, five plugs of *shadow-1* and four plugs of wild type were planted in the field in a 3-plug by 3-plug square. Plugs were randomly arranged within each square. Plugs were spaced 46 cm apart in each row, and 18 cm apart in each column. A plastic-wrapped cage was placed over the square to prevent undesired cross-pollination. Seeds were harvested separately from each plant on June 30, 2013, air-dried at room temperature, and storedat 4°C.

Two hundred *shadow-1* progeny seeds were planted in a 28 cm × 56 cm tray of Pro-Mix soil and were cold treated at 4°C for 2 weeks before being moved into the greenhouse. At the three-leaf stage, 200 individuals were randomly selected and transferred to plug trays, along with 10 wild-type and *shadow-1* M2 plants, and were allowed to grow for 2 months, after which height data were recorded. Progeny were divided into two groups: Non-dwarf progeny and dwarf progeny. Analysis of variance was performed on data collected from each set of plants using IBM SPSS 19.0 (IBM Corp., Somers, NY, USA). When sufficient differences (*P* < 0.05) were observed, Fisher’s protected least significant difference test (*P* = 0.05) was performed to calculate differences between groups. Six representative individuals were selected from the *shadow-1* dwarf progeny, in addition to six *shadow-1* M2 and six wild-type plants. These plants were vegetatively propagated in 50-plug trays as described above. Plants were maintained at a 5 cm height in full light for 6 weeks, and then placed in a 95% shade environment within the greenhouse for 2 weeks, after which photographs were taken.

### Application of GA_3_ to *shadow-1* Plants and TE to Wild-Type Plants

*shadow-1* and wild-type plants were vegetatively propagated in 50-plug trays as described above. Plants were maintained for 6 weeks after which they were cut down to a height of 5 cm. The plants were then separated into four groups, each containing six plugs of both *shadow-1* and wild type. The plants were sprayed with a GA_3_ solution, with different concentrations for each group (50, 100, and 150 mg/L, and water control). Plants were allowed to grow in the greenhouse under full light for 3 weeks, after which pictures and height data were taken. When testing GA_3_-treated *shadow-1* for shade tolerance, plants were prepared in the same manner as for full light GA_3_ application and separated into two groups. The first consisted of *shadow-1* plants treated with a 50 mg/L GA_3_ solution. The second consisted of wild-type plants treated with water, as a control. Three weeks after treatment, plants were cut to 5 cm and placed in a 95% shade environment within the greenhouse. After 2 weeks, photographs and height data were taken.

Wild-type and *shadow-1* plants were vegetatively propagated in 50-plug trays as described above. After 6 weeks, plants were cut to a height of 5 cm. Six plugs of both wild type and *shadow-1* were selected. Wild-type plants were treated with a 200 mg/L trinexapac-ethyl (TE) solution. *shadow-1* plants were treated with water, as a control. Plants were allowed to grow under full light in the greenhouse for 3 weeks, after which pictures and height data were taken. When testing TE-treated wild-type plants for shade tolerance, six wild type and *shadow-1* plugs were prepared and treated as described above. Three weeks after treatment, plants were cut to 5 cm and placed in a 95% shade environment within the greenhouse. After 2 weeks, photographs and height data were taken.

Analysis of variance was performed on height data collected from wild-type and *shadow-1* plants for both treatments as well as a non-treatment control under either full light or 95% shade using IBM SPSS 19.0 (IBM Corp., Somers, NY, USA). When sufficient differences (*P* < 0.05) were observed, Fisher’s protected least significant difference test (*P* = 0.05) was performed to calculate differences between groups.

### Quantification of GA_1_ Content

Wild-type and *shadow-1* plants were vegetatively propagated in 50-plug trays as described above and kept in the greenhouse. Plants were allowed to grow for 6 weeks before the experiment was initiated. Leaf samples were collected from wild type and *shadow-1* plants kept under either full light or 95% shade for 3 weeks. Leaf samples from 10 plants were pooled for each replicate. Two biological replicates were analyzed for each genotype and treatment. GA extractions were handled in the same manner as described for Kentucky bluegrass (*P. pratensis*) with modifications to include GA isoforms (Krishnan and Merewitz, 2015; Krishnan et al., 2016). About 200 mg of frozen leaf samples were ground to a fine powder in liquid nitrogen using a mortar and pestle. Prior to extraction, 100 nmol of deuterium-labeled GA_1_ was added as an internal standard for liquid chromatography (LC) analysis. GA_1_ content analysis was carried out using an ultra-high-performance LC-tandem mass spectrometer (UPLC/MS/MS) (Quattro Premier XE ACQUITY Tandem Quadrupole; Waters, Milford, MA, USA). Data were reported as a mean of two biological replicates. Analysis of variance was performed on GA_1_ content data collected from wild-type and *shadow-1* plants under both full light and 95% shade using IBM SPSS 19.0 (IBM Corp., Somers, NY, USA). When sufficient differences (*P* < 0.05) were observed, Fisher’s protected least significant difference test (*P* = 0.05) was performed to calculate differences between groups.

## Results

### Screening for Shade Tolerant Mutants

Gamma rays were used to mutagenize perennial ryegrass seeds, as previously described (Chen et al., 2016). Five kilograms of gamma-ray treated M1 seeds were planted in the field. The resultant plants were grown to maturity and allowed to randomly cross-pollinate in order to produce M2 seeds. In the fall of 2011, we first tested the efficacy of directly screening M2 seedlings for shade tolerance, using 150,000 M2 seeds. Seeds were germinated and seedlings were grown under 95% shade (~600 lux on a sunny day), which was produced by using black polyfiber cloth. Putative shade-tolerant seedlings were identified by de-etiolation, characterized by short coleoptiles, emergent true leaves, and an overall reduction in seedling height compared to wild type. A de-etiolated phenotype should be the result of seedling insensitivity to low light under our experimental conditions. We hypothesized that the insensitivity to low light expressed at the early seedling stage would result in mature plants’ insensitivity to shade. Consequently, some of the plant lines recovered should be shade tolerant. We identified 305 putatively shade-tolerant seedlings, which were allowed to recover under full light before they were again subjected to shade at maturity. Of the 305 plants tested, only four were confirmed to be shade tolerant through maturity (**Table 1**). Due to the low efficiency of our direct screen, we tested an alternative, two-step screening method for shade tolerance.

**Table 1:** The two-step screen is more effective than a direct screen at identifying shade-tolerant mutants of perennial ryegrass.

Screen	Time	Seeds used	Putative shade-tolerant mutants^a^/dwarf mutants^b^	Confirmed shade- tolerant mutants^c^
Direct screen	Fall, 2011	150,000	305	4 (1.3%)^d^
Two-step screen	Spring, 2012	150,000	51	29 (56.9%)
	Summer, 2012	150,000	85	36 (42.4%)

*M2 seedlings were used for both the direct screen and the two-step screen.*

*^a^For the direct screen, seeds were germinated under 95% artificial shade in the greenhouse, and seedlings with short coleoptiles, emergent true leaves, and overall reduced heights were considered putatively shade tolerant.*

*^b^For the two-step screen, dwarf screening was performed at the three-leaf stage, with dwarf mutants identified by a 30% reduction in height compared to wild type, dwarf plants were considered to be potentially shade tolerant.*

*^c^Confirmation of shade tolerance was performed on mature plants by placing them under 95% artificial shade in the greenhouse.*

*^d^Percentage of putative shade tolerant or dwarf seedlings that were confirmed to be shade tolerant.*

**FIGURE 2:**
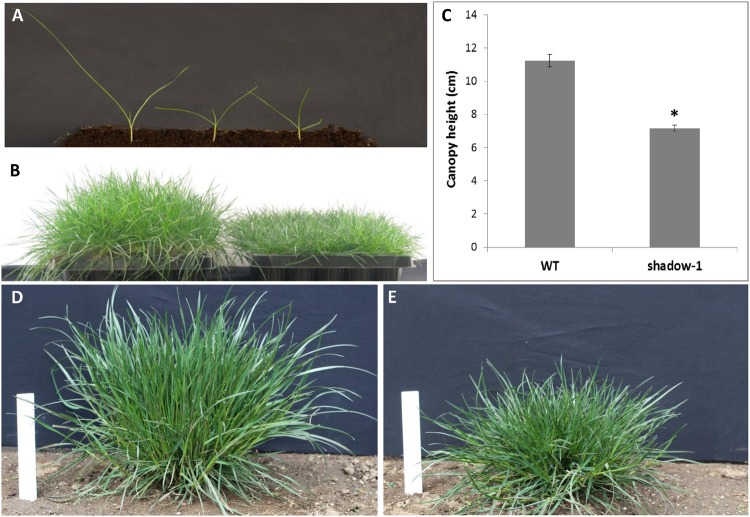
***shadow-1* plants have reduced canopy height compared to the wild-type perennial ryegrass (WT) under full light conditions. (A)** M2 seedlings were screened for dwarfism at the three-leaf stage. Dwarf plants (center and right) were identified as those with at least 30% reduction in plant height compared to WT (left). **(B)** Two-month-old, vegetatively propagated WT (left) and *shadow-1* plants (right). **(C)** Canopy heights of 2-month-old WT and *shadow-1* mutant plants. Data in **(C)** represent the mean of six replicates; each replicate was one representative plant. Bars represent the standard error. Asterisk represents a significant difference when compared to wild type using two-tailed Student’s *t*-test with pooled variance (*P* ≤ 0.05). **(D,E)** Field performance of WT **(D)** and *shadow-1* **(E).**

In the spring of 2012, we initiated our two-step screen on a population of M2 perennial ryegrass seedlings. M2 seedlings were visually screened for dwarfism at the three-leaf stage (~2 weeks after seeding). Dwarf mutants were identified by a ≥30% reduction in leaf height compared to wild type seedlings (**Figure 2A**). The screen identified 51 dwarf mutants from 150,000 M2 seeds, which were grown to maturity and then screened for shade tolerance. Of the 51 dwarf plants, 29 were identified as shade tolerant based on the absence of etiolation symptoms. The two-step screen was repeated over the summer of 2012 with an additional 150,000 M2 seeds, yielding 85 more dwarf mutants, 36 of which were shade tolerant (**Table 1**). Our direct screen for shade tolerance yielded four shade-tolerant mutant plants from 150,000 seedlings (0.003%) while the two-step method led to the recovery of 65 shade-tolerant mutants from 300,000 seedlings (0.022%).

### Evaluating Shade Tolerance of *shadow-1*

We chose one shade-tolerant mutant, *shadow-1*, for further evaluation under artificial and natural shade environments. In the greenhouse, *shadow-1* plants had significantly shorter canopy heights compared to wild type (**Figures 2B,C**). In the field, *shadow-1* maintained its dwarf phenotype, and showed no significant difference in tiller number or root:shoot biomass compared to wild type (**Figures 2D,E**; **Table 2**).

**Table 2:** *shadow-1* plants are dwarf compared to wild type under full light in the field.

Genotype	Canopy height (cm) (mean ± SE)	Tiller number (mean ± SE)	Dry root:shoot biomass (mean ± SE)
Wild type	19.84 ± 0.32	436.33 ± 31.67	0.21 ± 0.050
*shadow-1*	15.12 ± 0.45*	422.00 ± 40.50	0.22 ± 0.026

*Each value represents the mean of six replicates. Each replicate consisted of one representative plant. Measurements were taken on May 13, 2013, after 8 months in the field. Asterisks represent a significant difference when compared to wild type using two-tailed Student’s *t*-test (*P* ≤ 0.05). SE, standard error.*

**FIGURE 3:**
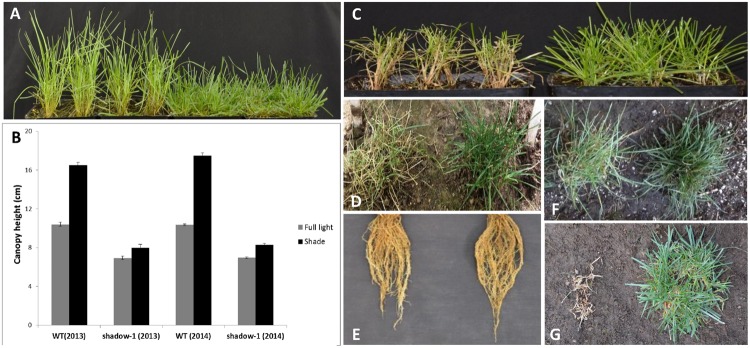
***shadow-1* plants display shade tolerance under both artificial- and natural-shade conditions. (A)** WT (left) and *shadow-1* plants (right) after 3 weeks under 95% artificial shade in the greenhouse. **(B)** Canopy heights of WT and *shadow-1* plants after 3 weeks under either full light or 95% artificial shade from two separate years. Data represent the average of six replicates. Each replicate consisted of the average height within each pen pack. Bars represent the standard error. **(C)** WT (left) and *shadow-1* plants (right) after 6 weeks under 95% artificial shade. **(D)** WT (left) and *shadow-1* (right) plants after 2 months under 95% natural shade in the field. **(E)** The root system of WT (left) and *shadow-1* (right) plants after 2 months under 95% natural shade in the field. **(F,G)** WT (left) and *shadow-1* (right) plants under 85% natural shade in the field after 1 month **(F)** and 30 months **(G)**.

We subjected *shadow-1* plants to 95% shade in the greenhouse. After 3 weeks, *shadow-1* displayed a distinct lack of etiolation, as indicated by healthy leaf color and reduced canopy height compared to wild-type controls (**Figures 3A,B**). Both sets of plants were maintained under shade for an additional 3 weeks. At the end of treatment, wild-type plants suffered severe damage while *shadow-1* plants sustained a healthy appearance, suggesting that the mutant plants were shade tolerant compared to wild type (**Figure 3C**).

*shadow-1* was planted in a densely wooded section of the field to determine how it would perform in a natural shade setting. Beginning in the spring, after shade reached 95% (~600 lux on a sunny day), we maintained plants at a 5 cm canopy height, cutting them whenever they reached 7.5 cm. Over a 2-month period, the growth rate of *shadow-1* was significantly reduced compared to wild type, as evidenced by a reduction in cutting frequency (**Table 3**). Under these conditions *shadow-1* plants displayed healthy growth, while the leaves of the wild-type plants suffered severe die off (**Figure 3D**). *shadow-1* plants had more tillers and greater root biomass than wild-type plants (**Figure 3E**; **Table 3**). Similar results were obtained when the experiment was repeated in the subsequent year.

**Table 3:** *shadow-1* plants outperform wild type under 95% natural shade in the field.

Genotype	Tiller number (mean ± SE)	Root biomass (g) (mean ± SE)	Cutting frequency (per month) (mean ± SE)
**Year 2013**			
Wild type	25.67 ± 2.52^a^	0.47 ± 0.03^a^	2.67 ± 0.67^a^
*shadow-1*	38.33 ± 1.53^b^	0.59 ± 0.05_b_	1.11 ± 0.39^b^
**Year 2014**			
Wild type	28.33 ± 2.08^a^	0.44 ± 0.05^a^	2.89 ± 0.39^a^
*shadow-1*	35.00 ± 5.57^b^	0.64 ± 0.03^b^	1.33 ± 0.39^b^

*Each value represents the mean of six replicates. Each replicate consisted of one representative plant. Data were collected in July of 2013 and 2014, after plants spent 10 months in the field. Values in the same column followed by the same letter are not significantly different from each other according to Fisher’s protected LSD (*P* = 0.05). SE, standard error.*

We evaluated the long-term performance of *shadow-1* under shade stress by planting *shadow-1* plants alongside wild type in 85% natural shade (~1800 lux on a sunny day). One month after planting, wild-type plants began to deteriorate, while mutant plants maintained healthy growth (**Figure 3F**). After 30 months (two winters), wild-type plants had completely died while *shadow-1* plants maintained green color and actually increased in overall size (**Figure 3G**).

### Confirming Dwarfism, Shade Tolerance, and Phenotypic Dominance in *shadow-1* Progeny

**FIGURE 4:**
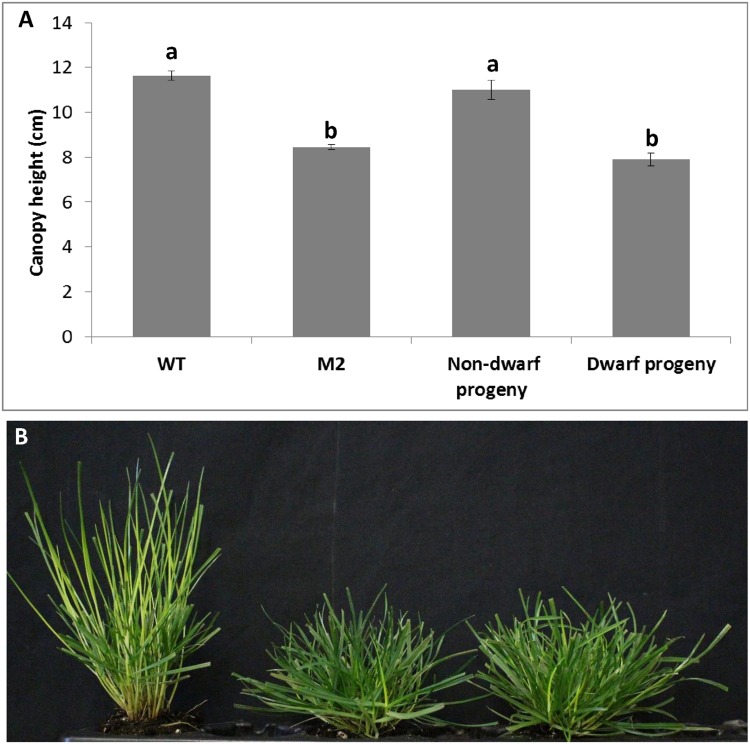
***shadow-1* plants successfully pass down dominant dwarfism and shade tolerance to progeny. (A)** Canopy heights of 2-month-old WT, *shadow-1* M2, Non-dwarf progeny, and Dwarf progeny under full light. Bars represent the standard error. Bars with the same letter above them are not significantly different from each other according to Fisher’s protected least significant difference (*P* = 0.05). **(B)** Appearances of WT (left), *shadow-1* M2 (center), and Dwarf progeny (right) after 2 weeks under 95% artificial shade. Non-dwarf progeny and dwarf progeny were from crosses between *shadow-1* M2 plants and wild-type plants.

We crossed *shadow-1* M2 plants with wild-type plants, generating a population of progeny plants, in order to determine the dominance of the mutation(s) causing the dwarf and shade tolerant phenotypes. We screened a random sample of 200 progeny plants produced from crosses between *shadow-1* M2 plants and wild-type plants and found that 106 were dwarf (53%), which demonstrated that dwarfism was dominant in *shadow-1* plants according to Mendelian inheritance patterns. The dwarf progeny were determined to have the same degree of dwarfism as M2 plants, while the non-dwarfs were of a similar height to wild type (**Figure 4A**). The dwarf progeny were also shade tolerant, showing the same level of shade resistance as *shadow-1* M2 plants (**Figure 4B**). These results demonstrate that the dwarf and shade tolerant phenotypes observed in *shadow-1* are most likely due to one, or more, dominant mutations.

### Determining Roles of GA in Dwarfism and Shade Tolerance of *shadow-1*

**FIGURE 5:**
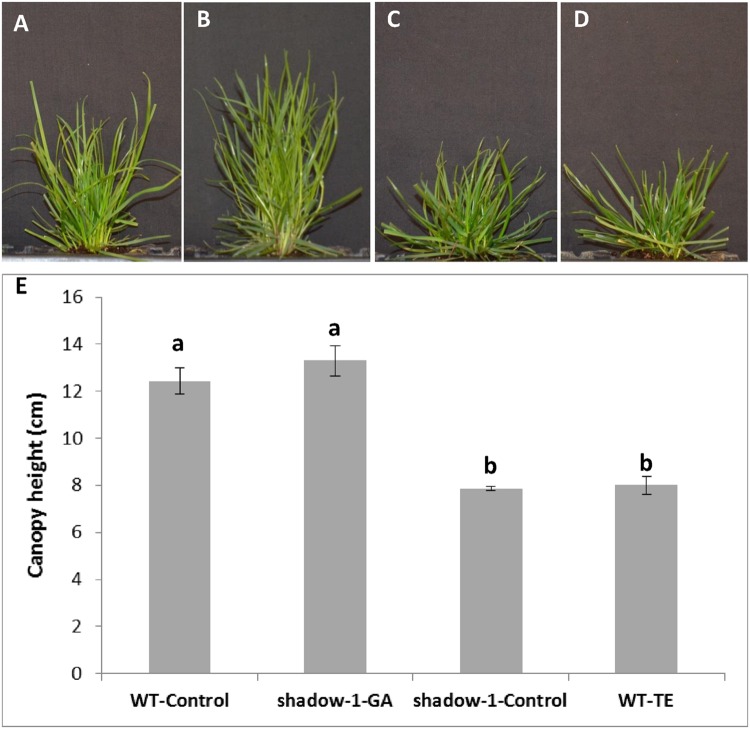
**Applications of gibberellic acid (GA_3_) to *shadow-1* and trinexapac-ethyl (TE) to WT reveal a connection between GA and dwarfism. (A)** WT plant grown under full light with no chemical treatment. **(B)** *shadow-1* plant grown under full light, treated with 50 mg/L GA_3_. **(C)** *shadow-1* plant grown under full light with no chemical treatment. **(D)** WT plant grown under full light, treated with 200 mg/L TE. **(E)** Canopy heights of treated and untreated WT and *shadow-1* plants. Data represent the average of six replicates under that treatment. Each replicate consisted of one plant. All photographs were taken 3 weeks after chemical application. Bars represent the standard error. Bars with the same letter above them are not significantly different from each other according to Fisher’s protected least significant difference (*P* = 0.05).

It has been well documented that many dominant dwarf mutants have defects in GA pathways (signaling or metabolic; Hedden, 2016), therefore we treated mutant plants with exogenous gibberellic acid (GA_3_) in an attempt to characterize the mechanism(s) behind *shadow-1*’s phenotypes. We conducted our exogenous GA treatment experiment by spraying wild type and *shadow-1* plants with a 50 mg/L GA_3_ solution. This dose was sufficient to restore the canopy height of *shadow-1* to that of untreated wild-type plants (**Figures 5A,B,E**). We also recreated a dwarf phenotype in wild-type plants through the application of a GA biosynthesis inhibitor, trinexapac-ethyl (TE). Wild type treated with TE showed a significant reduction in canopy height corresponding to the reduction observed in *shadow-1* (**Figures 5C–E**). *shadow-1* plants treated with TE showed no reduction in canopy height (data not shown). The results of these experiments suggest that GA deficiency might be responsible for the dwarf phenotype displayed by *shadow-1* plants.

**FIGURE 6:**
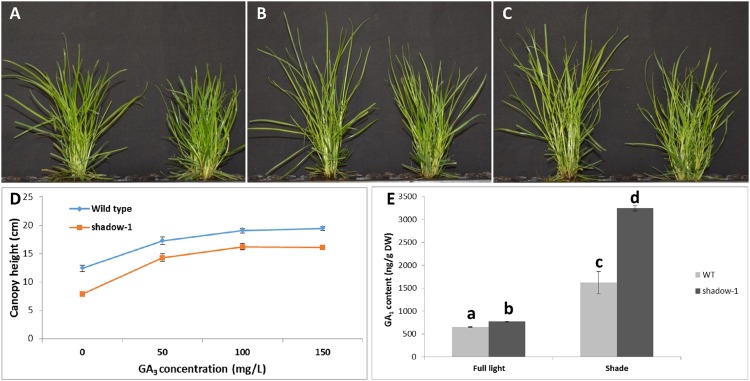
**Application of exogenous GA and detection of endogenous GA reveal GA insensitivity in *shadow-1* plants. (A–C)** WT (left) and *shadow-1* (right) plants treated with 50 mg/L GA_3_ **(A)**, 100 mg/L GA_3_ (**B**), or 150 mg/L GA_3_ **(C). (D)** Comparison of canopy heights for *shadow-1* and WT treated with varying concentrations of GA_3_. Each data point represents the average height of six replicates. Each replicate consisted of one plant. All photographs and data were taken 3 weeks after GA_3_ application. **(E)** GA_1_ content for *shadow-1* and WT under full light and 95% artificial shade conditions. Data represent the average of two replicates under each treatment. Each replicate consists of the pooled leaf samples from 10 plants. Bars represent the standard error. Bars with the different letter above them are significantly different from each other according to Fisher’s protected least significant difference (*P* ≤ 0.05).

To further study the involvement of GA in the phenotypes displayed by *shadow-1*, we treated both *shadow-1* and wild-type plants with one of a 50, 100, or 150 mg/L solution of GA_3._ The results are shown in **Figure 6**. We graphed the heights of *shadow-1* and wild-type plants for each treatment (**Figure 6D**). Doses of exogenous GA_3_ in excess of 100 mg/L had no additional effect on the heights of either *shadow-1* or wild-type plants. However, at all GA_3_ concentrations the canopy heights of *shadow-1* were significantly lower than those of the wild-type plants. In other words, the canopy heights of *shadow-1* could not reach those of the GA_3_-treated wild-type plants even at the highest GA concentration used (i.e., 150 mg/L), even though canopy heights of both wild type and *shadow-1* had individually plateaued (**Figure 6D**). These results demonstrate that *shadow-1* plants have a reduced response to GA compared to wild type, and thus GA insensitivity should play a partial role in the dwarfism exhibited by *shadow-1* plants.

We analyzed the endogenous GA_1_ content of both *shadow-1* and wild-type plants under both full-light and 95% shade conditions. GA_1_ is one of the main bioactive GAs in higher plants (Davière and Achard, 2013). Under both environments, the endogenous GA_1_ content of *shadow-1* was significantly higher than that of wild type (**Figure 6E**), demonstrating that *shadow-1* plants were not GA deficient. Interestingly, when subjected to shade stress, the relative level of endogenous GA_1_ within both *shadow-1* and wild type increased around fourfold and 2.5-fold, respectively (**Figure 6E**). GA insensitive plant mutants, such as the *gai-1* mutant of *Arabidopsis*, have also been reported to contain increased levels of endogenous GA (Peng et al., 1997). These results support the hypothesis that both of the *shadow-1* phenotypes are at least partially caused by GA insensitivity.

**FIGURE 7:**
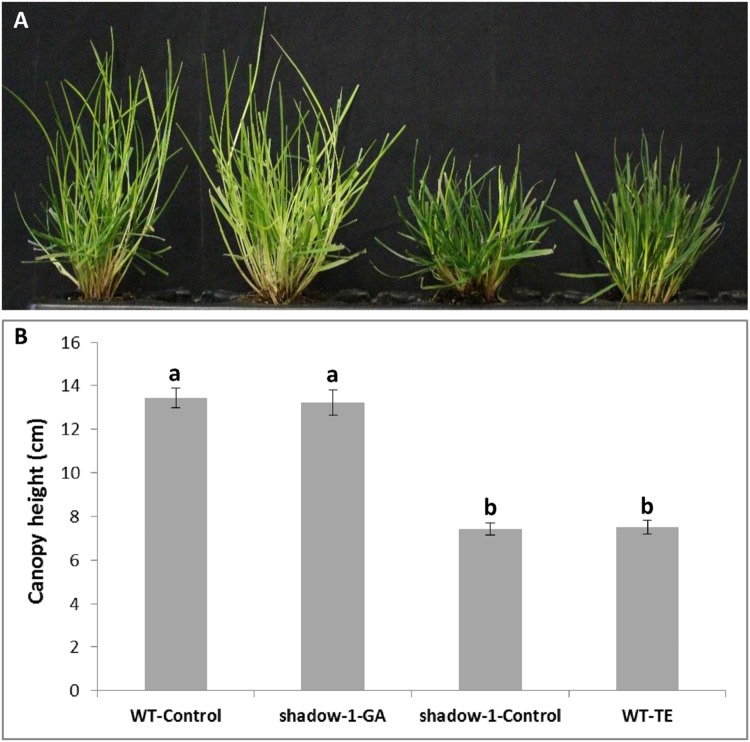
**Applications of gibberellic acid (GA_3_) to *shadow-1* and TE to WT demonstrate a link between shade tolerance and GA. (A)** Untreated WT (far left), GA_3_-treated *shadow-1* (center left), untreated *shadow-1* (center right), and TE-treated WT (far right) after 2 weeks under 95% artificial shade. **(B)** Canopy height comparisons between all lines and treatments shown in **(A)**. Plants were allowed to grow in full light for 3 weeks after chemical application before they were placed under artificial shade. All photographs were taken 2 weeks after shade treatment. Data represent the average of six replicates. Each replicate consisted of one plant. Bars represent the standard error. Bars with the same letter above them are not significantly different from each other according to Fisher’s protected least significant difference (*P* = 0.05).

We tested whether applications of exogenous GA to *shadow-1* plants could duplicate induce a wild-type shade response. After GA_3_ treatment, *shadow-1* plants lost shade tolerance – as evidenced by their yellow discoloration (**Figures 7A,B**). Conversely, when TE was used to block GA biosynthesis in wild-type plants, they gained shade tolerance comparable to *shadow-1* plants (**Figures 7A,B**). When we treated wild-type plants with exogenous GA, they became more sensitive to shade than the controls (data not shown). These results provide additional evidence that the dwarfism and shade tolerance displayed in *shadow-1* are due to impaired GA signaling.

## Discussion

Our results showed that directly screening perennial ryegrass mutants for shade tolerance was inefficient when performed on seedlings; therefore, screening should be performed at plant maturity. However, screening mature plants for shade tolerance entails the large-scale exposure of a mutant population to long-term (≥6 weeks) shade stress, which is cumbersome. The two-step screening method first screens for dominant dwarf mutants at the three-leaf stage (step 1) and then screens mature dwarf plants for shade tolerance (step 2). This method drastically reduces the number of mature plants required for shade tolerance screening because the large-scale dwarfism screen is performed when plants are small. The difference in space required for each screening method is dramatic, as 3000 seeds can fit inside a 0.162 m^2^ tray, as opposed to only 50 mature plants. Another challenge for direct screening is the unknown odds of success due to the lack of research about the development of shade tolerant mutant plants via mutation breeding. Screening for dwarfism in perennial ryegrass can easily be done under greenhouse conditions as previously described (Chen et al., 2016). With two-step screening, 136 dwarf-mutant lines were identified, 65 of which exhibited putative shade tolerance, accounting for about 48% of all recovered dwarf-mutant lines. This two-step screening method should be effective for developing new cultivars adapted to growth in reduced-light environments. Shade tolerant cultivars can expand the landscaping utility of turf, and other ground covers, to heavily wooded areas or areas with prohibitive amounts of shade-producing structures. For other high-value horticultural crops, these cultivars could vastly increase land-use efficiency, as producers could plant them under the canopies of existing vegetation, increasing available growing space.

By itself, dwarfism can be a highly desirable trait for turfgrass because dwarf cultivars require less frequent mowing and can therefore reduce costs associated with lawn maintenance (Johnson, 1994). There are other beneficial phenotypes associated with dwarfism in turfgrass, such as drought tolerance (Lu et al., 2009). Additionally, we have reported that, in perennial ryegrass, prostrate growth can be a beneficial secondary phenotype associated with dwarfism (Chen et al., 2016). Prostrate turf varieties are highly desirable because of their potentially increased heat resistance, traffic resistance, ground coverage, and tolerance to short mowing heights compared to upright varieties (Youngner, 1969).

Shade occurs on almost all lawns, and growing healthy lawns under shade conditions is often a challenge for both residential and commercial lawn owners (Koh et al., 2003). Shade can be formed by either artificial (e.g., buildings and awnings) or natural (e.g., trees) sources, with the latter contributing to a change in light quality in addition to an overall reduction of light intensity (Wherley et al., 2005; Takano et al., 2009). The *shadow-1* mutant line, isolated from our two-step screening method, is highly tolerant to extreme shade (85–95%) from both artificial and natural sources, suggesting that *shadow-1* can be used in commercial and residential lawns to reduce the negative impact of shade. *shadow-1* has reduced canopy height compared to wild type, and has normal root mass and tiller number. Reduction in canopy height can reduce mowing frequencies under both normal-light and shade conditions, reducing costs for both landscapers and homeowners. The sexually propagated progeny of *shadow-1* display the same degree of dwarfism and de-etiolation as parental plants, demonstrating that the mutation(s) responsible for these traits are dominant and therefore *shadow-1* plants could be readily incorporated into turf breeding programs.

Gibberellin has been shown to play a key role in regulating canopy height in monocots (Ervin and Koski, 1998; Ordonio et al., 2014; Ma and Huang, 2016). It is likely that the phenotypes exhibited by *shadow-1* are due to one or more mutations in the GA signaling or metabolic pathways. Our data indicate that the canopy heights of *shadow-1* plants can be artificially restored to those of wild-type controls through the application of exogenous GA_3_, suggesting that *shadow-1* mutant plants may be GA deficient. However, detailed analyses demonstrate that, following exogenous GA_3_ application, *shadow-1* canopy heights were significantly lower than those of the wild type at the three GA_3_ concentrations used. Even at the highest GA_3_ concentration used, after the canopy heights plateaued, there remained a height difference between *shadow-1* and wild-type plants. These results provide strong evidence that the dwarfism observed in *shadow-1* is most likely due to a partial insensitivity to GA.

Determination of endogenous GA content in mutants revealed that mutant plants had higher bioactive GA (GA_1_) contents than wild-type plants. The phenomenon of higher endogenous bioactive GA contents in GA-insensitive dwarf mutants has been reported in a number of other plant species, including *Arabidopsis*, maize (*Zea mays* L.), and wheat (*Triticum aestivum* L.) (Talon et al., 1990; Dill et al., 2001), which is consistent with what we have observed in *shadow-1* mutant plants. These data further support the hypothesis that GA insensitivity is responsible for the dwarfism of *shadow-1* mutants. Our data also show that exogenous GA application can reduce shade tolerance in *shadow-1* mutants. Meanwhile, wild-type perennial ryegrass treated with TE, a GA biosynthesis inhibitor, gained shade tolerance similar to that observed in *shadow-1*. All of these data demonstrate that GA can be an important factor for shade tolerance. In future studies, we will conduct transcriptome sequencing analysis and hope to obtain the information regarding the possible relationship between the shade tolerance and GA insensitivity. We understand that GA is not the only factor for dwarfism or shade tolerance. Mutations in many genes including the ones involved in biosynthesis/catabolism or signaling pathways for auxin, cytokinins, or brassinosteroids may also lead to dwarfism. It is also possible that some of these non-GA mutants may be shade tolerant. The method we presented can also be used to isolate non-GA related shade tolerant mutants as if they display dwarfisms.

## Conclusion

Our novel two-step method for isolating plants with shade tolerance is straightforward and highly effective, and should be applicable to other crop plant species, including ground covers, and the high-value horticultural crops. This can be particularly important to species that do not currently have shade tolerant cultivars. Shade tolerant cultivars can be used under the canopies of existing vegetation to boost agricultural productivity per unit area of land. As shown by *shadow-1*, shade tolerance should be dominant in plants isolated from the two-step screening method, therefore these plants can be easily incorporated into perennial ryegrass breeding programs.

## Author Contributions

WL and LK-G did most of the work for mutant characterization. WL and CT were responsible for initial isolation of mutants. RE-T and HY were also involved greenhouse and field evaluation of the mutant. CT was involved in gamma-ray irradiation and producing M2 seeds and initial screening of the mutant. SK and EM were responsible for endogenous hormone analysis and manuscript editing. KG, JI, RM, and ZL provided advices on characterization of the mutant and manuscript editing. YL designed experiments for the mutant isolation and characterization. WL, LK-G, and YL were involved in manuscript writing and editing.

## Conflict of Interest Statement

The authors declare that the research was conducted in the absence of any commercial or financial relationships that could be construed as a potential conflict of interest.
